# SEOM Clinical Guideline of fertility preservation and reproduction in cancer patients (2016)

**DOI:** 10.1007/s12094-016-1587-9

**Published:** 2016-11-28

**Authors:** M. Muñoz, A. Santaballa, M. A. Seguí, C. Beato, S. de la Cruz, J. Espinosa, P. J. Fonseca, J. Perez, T. Quintanar, A. Blasco

**Affiliations:** 1 Servicio de Oncología Médica, Translational Genomics and Targeted Therapeutics in Solid Tumors, IDIBAPS, Hospital Clínic de Barcelona, Villarroel, 170-08036 Barcelona, Spain; 2 Hospital Universitari I Politècnic la Fe, Valencia, Spain; 3 Corporació Sanitària Parc Taulì, Sabadell, Spain; 4 Hospital Universitario Virgen Macarena, Seville, Spain; 5 Complejo Hospitalario de Navarra, Pamplona, Spain; 6 Hospital General de Ciudad Real, Ciudad Real, Spain; 7 Hospital Universitario Central de Asturias, Oviedo, Spain; 8 Hospital General Universitario de Elche y Vega Baja, Elche, Spain; 9 Hospital General Universitario de Valencia, Valencia, Spain

**Keywords:** Cancer patients, Fertility preservation, Cryopreservation, Infertility, Adolescent/child cancer

## Abstract

Chemotherapy and radiotherapy often result in reduced fertility in cancer patients. With increasing survival rates, fertility is an important quality-of-life concern for many young cancer patients. Around 70–75% of young cancer survivors are interested in parenthood but the numbers of patients who access fertility preservation techniques prior to treatment are significantly lower. Moreover, despite existing guidelines, healthcare professionals do not address fertility preservation issues adequately. There is a critical need for improvements in clinical care to ensure patients are well informed about infertility risks and fertility preservation options and to support them in their reproductive decision-making prior to cancer treatment.

## Introduction

When a patient is facing with cancer, the main concern is, definitively, the cure of the disease. However, the increasing large number of patients and long-term survivors oblige to discuss with them, before treatment would have been initiated, the possibility of losing fertility after cancer therapies. In this guideline, we will present global trend in this field, the consequence of surgery, radiotherapy or chemotherapy, and the main strategies to avoid sterility in patients, with the pros and cons of different methods.

## Cancer in young people

Patients aged 15–39 years old at the diagnosis constitute the adolescent and young adult cancer survivorship population, which includes approximately 700,000 patients diagnosed each year, or 2% of all invasive cancers diagnosed in the United States [1]. In Europe, an estimated 130,500 new cancer diagnoses (non-melanoma skin cancers being excluded) are made per year in this subset [2]. The most common types of cancer occurring in this group included: lymphoma (21%), melanoma (15%), cancer of the male genital system (11%), cancer involving the endocrine system (11%) and cancer of the female genital tract (9%).

Cancer therapy often affects reproductive organs, leading to impaired pubertal development, hormonal regulation, fertility, and sexual function, affecting quality of life. Table 1 shows the increased risk of infertility; however, the ultimate impact on reproductive potential depends on the age of the patient, the type, dose and duration of treatment, and the idiosyncrasies of the individual and the cancer [3, 4].

**Table 1:** Conditions with increased risk of infertility

Risk of infertility	Males	Females
High risk (>80% risk of permanent amenorrhea in women; prolonged azoospermia in men)	Radiation >2.5 Gy to testis Chlorambucil (1.4 g/m^2^) Cyclophosphamide (19 g/m^2^) Procarbazine (4 g/m^2^) Melphalan (140 mg/m^2^) Cisplatin (500 mg/m^2^) BCNU (1 g/m^2^) and CCNU (500 mg/m^2^)	Hematopoietic stem cell transplantation with cyclophosphamide Total-body irradiation or cyclophosphamide/busulfan External beam radiation to a field that includes the ovaries CMF, CEF, CAF, TAC × 6 cycles in women ≥40 years
Intermediate risk (40– 60% risk of permanent amenorrhea in women; likelihood of azoospermia in men)	Busulfan (600 mg/kg) Ifosfamide (42 g/m^2^) BCNU (300 mg/m^2^) Nitrogen mustard Actinomycin D	BEACOPP CMF, CEF, CAF, TAC × 6 cycles in women age 30–39 AC × 4 cycles in women ≥40 years AC or EC × 4 → Taxanes

*AC* adriamycin, cyclophosphamide, *BEACOPP* Bleomycin, Etoposide , Adriamycin, Cyclophosphamide, Vincristine, Procarbazine, Prednisona, *CAF* Cyclophosphamide , Adriamycin, 5-fluoruracil, *CEF* Cyclophosphamide, Epirrubicin, 5-fluoruracil, *CMF* cyclophosphamide, Methotrexate, 5-fluoruracil, *TAC* Docetaxel, Adriamycin, Cyclophosphamide

## Fertility preservation interventions, is it a demand of cancer patients?

All patients of reproductive age diagnosed with cancer should be informed of the potential for gonadal toxicity and the options to preserve future fertility. Cancer-related infertility has a negative influence on the quality of life [5]. International guidelines recommend previous treatment discussion with the patient about infertility, fertility preservation options, and needs for contraception prior to initiating therapy [6]. However, this practice has not become routine and the number of patients receiving this information is insufficient [5, 6].

Patients who choose the option of fertility preservation should be referred to appropriate reproductive specialists within 24 h and to a mental health professional to assist with complex decision-making if they needed [5]. A follow-up appointment should also be offered once the patient has completed treatment to allow discussion and information about the clinical use of stored gametes (if appropriate) [3, 4].

*Considering the above, discussion about their risk of infertility prior to initiating cancer treatment is a mandatory intervention for patients of reproductive age* (Evidence **IA**).

## Fertility preservation options for local treatment of cancer

### Fertility preservation options in patients undergoing radiotherapy

The gonads are very sensitive to radiotherapy, especially in prepubertal. Cranial irradiation also may induce infertility by disruption of the hypothalamic-pituitary-gonadal axis and disturbance normal hormonal secretion. [7]. Table 2 shows the risk of infertility after radiotherapy. Shielding of the gonadal area is the standard procedure for reducing scatter radiation to the reproductive organs and to preserve fertility. In pelvic irradiation, surgical ovarian transposition has been shown to reduce the risk of ovarian failure, in adult patients. Other cryopreservation techniques may be considered prior to radiotherapy administration [7].

**Table 2:** Risk of prolonged azoospermia in males or amenorrhea in females after radiotherapy

High risk	Intermediate risk
Total-body irradiation for bone marrow transplant/stem cell transplant Testicular radiation dose >2.5 Gy in adult men Testicular radiation dose ≥6 Gy in prepubertal boys Pelvic or whole abdominal radiation dose ≥6 Gy in adult women Pelvic or whole abdominal radiation dose ≥10 Gy in postpubertal girls Pelvic or whole abdominal radiation dose ≥15 Gy in prepubertal girl	Testicular radiation dose 1–6 Gy from scattered pelvic or abdominal radiation Pelvic or whole abdominal radiation dose 5–10 Gy in postpubertal girls Pelvic or whole abdominal radiation dose 10–15 Gy in prepubertal girls Craniospinal radiotherapy dose ≥25 Gy

Modified from Rodriguez-Wallberg et al. [7]

### Fertility preservation options in patients undergoing surgery

Surgical techniques for preserving reproductive systems without compromising survival are relatively recent and procedures are still evolving. Indications for conservative surgery include generally well-differentiated, low-grade tumor in its early stages, or low malignant potential [8]. Table 3 shows the options to fertility-sparing interventions in female patients.

**Table 3:** Options to fertility-sparing interventions in female patients undergoing surgery

Type of tumor	Surgery	Oncologic outcomes	Obstetric outcomes
Borderline ovarian tumors FIGO stage I	Unilateral oophorectomy	Oncologic outcome is comparable with the more radical approach of removing both ovaries and the uterus Recurrence 0–20 versus 12–58% when only cystectomy was performed	Pregnancies have been reported with a favorable obstetric outcome
Ovarian epithelial cancer stage I, grade 1	Unilateral oophorectomy	7% recurrence of the ovarian malignancy and 5% deaths	Pregnancies have been reported with a favorable obstetric outcome
Malignant ovarian germ cell tumors/sex cord stromal tumors	Unilateral oophorectomy	Risk of recurrence similar to historical controls	Pregnancies have been reported and favorable obstetric outcome
Cervical cancer stage IA1, 1A2, 1B1	Radical vaginal trachelectomy	Rates of recurrence and mortality are comparable with those described for similar cases treated by radical hysterectomy or radiation therapy	Spontaneous pregnancies described in up to 70%. Risk of second-trimester pregnancy loss and preterm delivery
Endometrial adenocarcinoma grade 1, stage 1A (without myometrial or cervical invasion)	Hormonal treatment with progestational agents for 6 months	Recurrence rate 30–40%; 5% recurrence during progesterone treatment	Pregnancies have been reported

Modified from Rodriguez-Wallberg et al. [7]

## Fertility preservation when coping with systemic treatment

### Toxicity of different schedules of chemotherapy and hormonotherapy

Regimens used in cancer patients provoke gonadal toxicity in both sexes. In adult women, the possibility of offspring is particularly low, especially in hematological and breast cancer survivors [4, 6, 9–11]. Patients with breast cancer have the lowest chance of subsequent pregnancy, about 70% compared to the general population [13, 15]. Gonadal toxicity depends on age and chemotherapy schedule received [9–12, 14, 15] (Table 4).

**Table 4:** Risk of infertility in different chemotherapy agents

Risk	Females	Males
Monotherapy		
High risk	Busulfan >600 mg/m^2^ Cyclophosphamide >7.5 g/m^2^ Chlorambucil Procarbazine	Busulfan 600 mg/m^2^ Chlorambucil 1.4 g/m^2^ Cyclophosphamide 19 g/m^2^ Procarbazine 4 g/m^2^ Melphalan 140 mg/m^2^ Cisplatin 500 mg/m^2^ If treatment occurred prior to puberty: BCNU (Carmustine) 1 g/m^2^ CCNU (Lomustine) 500 mg/m^2^
Medium risk	Cisplatin Doxorubicin Bevacizumab (34% ovarian failure)	With other highly sterilizing agents: Ifosfamide 42 g/m^2^ BCNU 300 mg/m^2^ Nitrogen mustard Actinomycin D
Low risk	Vincristine Methotrexate	Temporary reduction in sperm count: Adriamycin 770 mg/m^2^ Thiotepa 400 mg/m^2^ Cytosine arabinoside 1 g/m^2^ Vinblastine 50 mg/m^2^ Vincristine 300 mg/m^2^ Amsacrine, bleomycin, dacarbazine, daunorubicin, epirubicin, etoposide, fludarabine, 5-fluorouracil, 6-mercaptopurine, methotrexate, mitoxantrone, thioguanine
Polychemotherapy		
High risk	AC × 4, >40 years, 57–63% CMF × 6, >40 years, 79–96% CAF/CEF × 6, >40 years, 60–90% escBEACOPP, >35 years, >50% BEAMcam, >40 years, 75%	BEACOPP >80%
Medium risk	CMF × 6, 30–40 years, 31–38% CAF/CEF × 6, <30 years, 23–47% DA-R-EPOCH, <35 years, 30%	CHOP × 6
Low risk	AC × 4, 30–40 years, 13% CMF × 6, <30 years, 19% ABVD CHOP × 6	ABVD

Modified from several sources: Azim et al [37], Meistrich [15]

### Methods to preserve fertility in males with cancer

The options for preservation of fertility in males include [16, 17]:

1. **Sperm cryopreservation** [18]**:** *semen cryopreservation with one to three samples collection is recommended* (Evidence **IIIA**). Sperm collection is recommended before star treatment, because there is a potential risk. Methods such as intracytoplasmic sperm injection [19] allow the future use of a very limited amount of sperm. When patients are unable to ejaculate, alternative methods such as urine collection after retrograde ejaculation, rectal electroejaculation under anesthesia and testicular sperm aspiration are an option.
2. **Hormonal gonadoprotection:** *hormonotherapy in men is not recommended* (Evidence **IIID**).
3. **Other methods:** testicular tissue or spermatogonial cryopreservation and reimplantation or grafting of human testicular tissue are still experimental. However, these approaches may be the only option of fertility preservation available to prepubertal boys.

### Methods to preserve fertility in females with cancer

Different methods to preserve fertility could be proposed depending on patients’ age, current success rate, required delay in cancer treatment, required ovarian stimulation, sperm requirement and risk of reintroducing malignant cells (Table 5) [20–22]:

**Table 5:** Methods to preserve fertility in females with cancer

Methods	¿Need ovarian stimulation?	¿Delay cancer treatment?	¿Need male partner or sperm donor?	Success rates	Special considerations
GnRHa	No	No	No		Controversial, just partially recommended in ER-negative breast cancer patient
Embryo freezing	Yes	Yes	Yes	Cumulative pregnancy rate of 66% among women with cancer	
Oocyte cryopreservation	Yes	Yes	No	Pregnancy rate per cycle of 50.2% or per embryo transfer 55.4%	
Immature oocyte cryopreservation	No	No	No		
Ovarian tissue cryopreservation	No	No	No	Pregnancy rate of 25% among women with cancer	No indication when high risk of ovarian metastases

1. **Embryo cryopreservation:** *embryo cryopreservation is a widely established method for female fertility preservation*, with a reported life birth rate of 44.4% (Evidence **IIA**).
2. **Oocyte cryopreservation:** *this is the preferent option when embryo cryopreservation is not possible*, particularly for patients without a male partner or have religious or ethical objections to embryo freezing (Evidence **IIA**). Oocyte cryopreservation can be done either by conventional slow freezing or by vitrification. Vitrification is now the most widely used method of oocyte cryopreservation due to the improved survival and fertilization rates, compared to slow freezing method [23, 24].
3. **Immature oocyte cryopreservation:** the major limitation of embryo and mature oocyte cryopreservation is the time to complete ovarian stimulation. Immature oocyte cryopreservation is not as successful as cryopreserving oocytes or embryos that have matured in vivo and is considered experimental.
4. **Ovarian tissue cryopreservation and transplantation:** ovarian tissue cryopreservation involves harvesting and freezing ovarian tissue, allowing preservation of oocytes within primordial follicles. In the future, the tissue can be autotransplanted or matured in vitro. This procedure allows immediate initiation of cancer treatment as it does not require prior ovarian stimulation nor sperm donation. To date, there have been at least 60 live births after *ovarian tissue transplantation* [25] (Evidence **IIIB**).
5. **Ovarian protection with gonadotropin-releasing hormone agonists (GnRHa):** the role of GnRHa to preserve ovarian function during chemotherapy has been investigated in many randomized trials and 14 meta-analyses have been published [26]. Regarding pregnancy outcomes, only nine meta-analyses investigated this question and only three studies found a positive impact of GnRHa on pregnancy outcomes. The Prevention of Early Menopause Study (POEMS) randomized 218 premenopausal women with early-stage, receptor-negative breast cancer. The study demonstrated a reduced incidence of ovarian failure and higher rates of pregnancy with the administration of GnRHa. However, other study reviewed the efficacy of GnRHa use in lymphoma patiens [27] but not benefit could be demonstrates. *The use of GnRHa could be an option to discuss with patients with early*-*stage receptor negative breast cancer if embryo or oocyte cryopreservation not feasible* (Evidence **IIB**). *The use of GnRHa to preserve fertility in women with other cancer should not be recommended* (Evidence **IIB**).

## Special concerns in breast and ovarian cancer

Women diagnosed with breast cancer have the lowest chance of subsequent pregnancy which is nearly 70% lower compared to the general population. This is believed to be secondary to frequent treatment with gonadotoxic chemotherapy, prolonged treatment periods with tamoxifen in patients with endocrine-sensitive disease and also a general misconception that pregnancy could stimulate cancer recurrence being a hormonally driven disease [28].

Based on accumulating research, there does not appear to be an increased risk of cancer recurrence in women as a result of fertility preservation and pregnancy, including those with hormonally sensitive tumors [29]. Conventional ovarian stimulation causes supra-physiological estradiol (E2) levels and, therefore, may be unsuitable for women with estrogen receptor-positive (ER+) tumors. Instead, ovulation induction regimens incorporating tamoxifen or aromatase inhibitors (AIs) can be used resulting in attenuated E2 levels without compromising embryo or oocyte viability. Additional fertility counseling for women who have a BRCA1 or 2 mutation (or are at high risk of having) is warranted to educate them about the available fertility preservation options in the context of their unique concerns. Women with BRCA mutations may elect to use preimplantation genetic diagnosis during in vitro fertilization to avoid transmitting the mutation [30] (Table 6).

**Table 6:** Pregnancy in LSV

Situation	Proposed action
Increase risk of immediate and later health complications	Cancer patients should be informed before starting anticancer treatment
Potential risk of pregnancy for themselves and their offsprings	Patients should be aware about risk of cancer recurrence, difficulty in early cancer detection during pregnancy and hereditary syndromes
Increased risk of miscarriages in patients with pelvic irradiation	Stop smoking, because increase incidence or miscarriages in this situation
No different physical conditions or about life style have been related with adverse pregnancy outcomes	No definitive dates. Research should be continued
High dose of alkylating drugs and cisplatin	Decreased likelihood of siring a pregnancy in male survivors of childhood cancer
Incidence of potential obstetric and offspring risks of morbid conditions associated with anticancer treatment as well as fertility preservation options in cancer survivors	Medical professionals should be properly trained

About 7% of epithelial ovarian cancer (EOC) patients are diagnosed under 40 and 2% of those are even younger than 30 years of age. In young patients having desire to preserve fertility, conservative strategies could be applied, consisting in conservative surgical approaches as unilateral salpingo-oophorectomy on the side of the tumor and optimal surgical staging [31]. The majority of guidelines suggest a conservative approach in stage IA–B and grade 1 tumors and non-clear cell histology. Data regarding stage IC or stage IA–B grade III are too far limited. Conservative surgery should be avoided in stage IB and IC tumors with bilateral involvement.

Malignant ovarian germ cell tumors (MOGCT) represent 5% of all ovarian neoplasms. Cisplatin-based regimens are now preferred because they seem to offer a better fertility outcome than non-cisplatin-based chemotherapies. Fertility-sparing surgery may be the standard treatment in young patients with early-stage BOTs [32].

## Preservation Ferlitity Options in Prepubertal Patients

There are new observational and case studies addressing fertility preservation of children and adolescents with cancer, including the risks of radiation as well as chemotherapy. Current techniques are limited by the patient’s sexual immaturity and all available approaches for children are experimental [17].

### Females

For prepubertal patients, we recommend to use established methods of fertility preservation (gonadal tissue cryopreservation, radiation shielding or ovarian transposition), with patient assent and parent consent [17].

Ovarian tissue cryopreservation followed by heterotopic implantation (e.g., in the abdominal wall, forearm, chest wall) or orthotopic implantation (e.g., to remaining ovarian tissue or pelvic peritoneum), after the end of oncologic treatment, is an investigational approach. *No reports of live births after ovarian cortical tissue cryopreserved prepubertally and reimplanted at a later date, primarily because of the young age of the study participants* [33] (Evidence **IVB**).

### Males

Due to the fact that active spermatogenesis only starts from puberty onwards, prepubertal boys cannot benefit from sperm cryopreservation. A potential alternative strategy for preserving their fertility involves storage of immature gametes and gonadal stem cells after testicular tissue sampling in the hope that future technologies will allow its safe utilization [18, 34]. *Although prepubertal testicular stem cell banking is being introduced into clinical practice, this approach should be regarded as experimental in view of the paucity of evidence of successful transplantation and the scarce safety data for this method* [18] (Evidence **VB**). *There is no role for gonadal protection by any form of hormonal or pharmacological means in either boys or girls* [16] (Evidence **IIIB**).

### Pregnancy in LSV: recommendations

Albeit studies analyzing the effect of previous anticancer treatments (chemotherapy, radiotherapy, surgery and/or hematopoietic stem cell transplant) on pregnancy and livebirth are not enough, important reviews have been published (Table 6). One of them [10] observed that greater doses of contemporary alkylating drugs and cisplatin were associated with a decreased likelihood of siring a pregnancy in male survivors of childhood cancer.

Other investigator’s group [35] evaluates the associations between pre-pregnancy lifestyle factors, psychologic distress, and adverse pregnancy outcomes in a large cohort of 1192 female survivors of childhood cancer. This study concluded that the risk for miscarriage was significantly increased among survivors exposed to >2.5 Gy uterine radiation who had a history of smoking.

Tarín et al. [36] observed that exist different morbid conditions associated with anticancer therapies, as thyroid dysfunction, hyperprolactinemia, hyperglycemia or hypertension, may be risk factors for pregnancy or offspring.

Diagnosis and treatment of thyroid dysfunction before/during pregnancy may prevent or alleviate the effects of maternal thyroid disease on early brain development. Likewise, untreated hyperprolactinemia may be a risk factor for ectopical pregnancy.

Maternal age at childbirth is steadily rising in many Western populations, and female cancer survivors are not an exception to this general trend. The resulting obstetric and offspring risks associated with postponed maternity may be superimposed on those already present in cancer survivors.
